# Role of the Epicardium in the Development of the Atrioventricular Valves and Its Relevance to the Pathogenesis of Myxomatous Valve Disease

**DOI:** 10.3390/jcdd8050054

**Published:** 2021-05-12

**Authors:** Renélyn Wolters, Ray Deepe, Jenna Drummond, Andrew B. Harvey, Emilye Hiriart, Marie M. Lockhart, Maurice J. B. van den Hoff, Russell A. Norris, Andy Wessels

**Affiliations:** 1 Department of Regenerative Medicine and Cell Biology, Medical University of South Carolina, 173 Ashley Avenue, Charleston, SC 29425, USA; woltersr@musc.edu (R.W.); deepe@musc.edu (R.D.); drummonj@musc.edu (J.D.); harveyan@musc.edu (A.B.H.); hiriart.emilie@gmail.com (E.H.); marie.lockhart@gmail.com (M.M.L.); norrisra@musc.edu (R.A.N.); 2 Department of Medical Biology, Amsterdam UMC, Academic Medical Center, Meibergdreef 15, 1105 AZ Amsterdam, The Netherlands; m.j.vandenhoff@amsterdamumc.nl

**Keywords:** atrioventricular valve, epicardium, lateral cushion, major cushion, myxomatous degeneration

## Abstract

This paper is dedicated to the memory of Dr. Adriana “Adri” Gittenberger-de Groot and in appreciation of her work in the field of developmental cardiovascular biology and the legacy that she has left behind. During her impressive career, Dr. Gittenberger-de Groot studied many aspects of heart development, including aspects of cardiac valve formation and disease and the role of the epicardium in the formation of the heart. In this contribution, we review some of the work on the role of epicardially-derived cells (EPDCs) in the development of the atrioventricular valves and their potential involvement in the pathogenesis of myxomatous valve disease (MVD). We provide an overview of critical events in the development of the atrioventricular junction, discuss the role of the epicardium in these events, and illustrate how interfering with molecular mechanisms that are involved in the epicardial-dependent formation of the atrioventricular junction leads to a number of abnormalities. These abnormalities include defects of the AV valves that resemble those observed in humans that suffer from MVD. The studies demonstrate the importance of the epicardium for the proper formation and maturation of the AV valves and show that the possibility of epicardial-associated developmental defects should be taken into consideration when determining the genetic origin and pathogenesis of MVD.

## 1. Introduction

Myxomatous degeneration involving the leaflets of the left atrioventricular valve (mitral valve) leads to myxomatous valve disease (MVD), a common cause of Mitral Valve Prolapse (MVP). MVP affects nearly 1 in 40 individuals [1,2,3,4,5,6] and is a serious condition characterized by abnormal systolic “bulging” or “billowing” of leaflets of the mitral valve into the left atrium, a situation typically accompanied by mitral regurgitation [7]. There are no therapies known to improve or reverse the valve abnormality, and surgical and catheter-based approaches to improve valve function are invasive and expensive. MVP can be found in a variety of conditions, including rare syndromic diseases, such as Marfan syndrome (MFS), Loeys–Dietz syndrome (LDS), Aneurysm-osteoarthritis syndrome (AOS), Williams–Beuren syndrome (WBS), and Ehlers–Danlos syndrome (EDS) [6]. Non-syndromic MVP is, however, more common. Insight into the genetic etiology of familial non-syndromic MVP has been obtained from studying pedigrees of families in which multiple individuals have MVD. This approach has thus far led to the identification of a small number of causal genes, including FILAMIN-A (FLNA) [1,2,4] and Dachsous (DCHS1) [3]. Despite these advancements, relatively little is known about the molecular and cellular mechanisms involved in MVP pathogenesis. It is interesting to note that while MVP is the most serious and widespread AV valve abnormality, tricuspid valve prolapse is observed in up to 50% of patients with primary or non-syndromic MVP [8,9]. In this contribution, we will review the developmental events that underlie the formation of the respective leaflets of the atrioventricular (AV) valves, specifically focusing on the role of the epicardium in this specific event. We will show that epicardium/epicardially-derived cells (EPDCs) [10] are not only playing a crucially important role for the development of the AV junction and AV valves, but also that molecular mechanisms that govern the regulation of epicardial development in this part of the heart may need more attention in efforts to elucidate the underlying mechanisms leading to MVD/MVP at later stages of life.

## 2. The Atrioventricular Cushions

**Figure 1:**
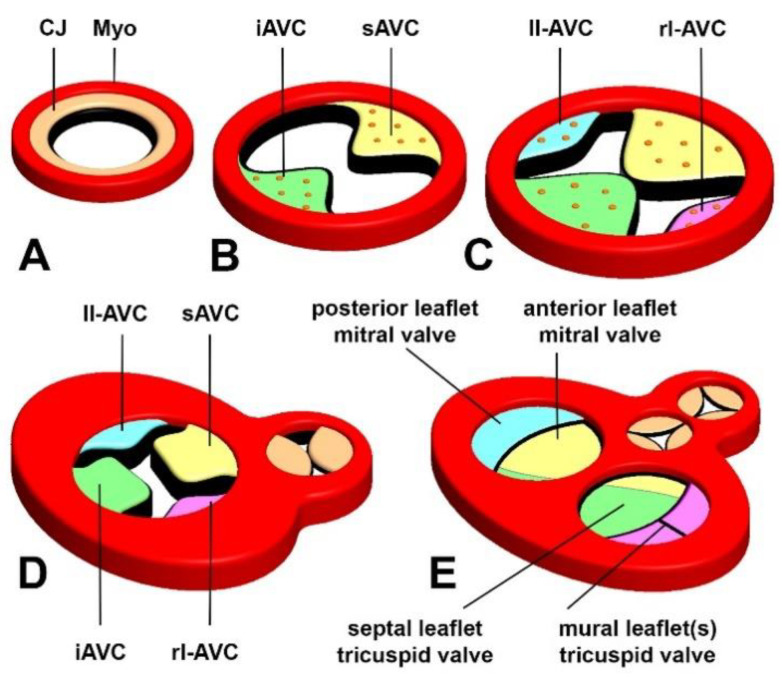
Origin and Fate of the AV cushions. This illustration reflects the respective stages of AV cushion formation. (**A**) Cardiac jelly accumulates between myocardium and endocardium in the tubular heart, (**B**) formation of the major AV cushions, (**C**) fusion of the major cushions and formation of the lateral cushions. (**D**,**E**) illustrate how the respective cushion contribute to the leaflets of the formed heart. CJ, cardiac jelly; Myo, myocardium; iAVC, inferior AV cushion; sAVC, superior AV cushion; ll-AVC, left lateral AV cushion; rlAVC, right lateral AV cushion, muMiV, mural leaflet of the mitral valve.

Shortly after the formation of the primary heart tube, an extracellular matrix (ECM)-rich substance, often referred to as the cardiac jelly, can be found between the myocardium and endocardium (Figure 1). As the heart tube starts to loop, further accumulation of the cardiac jelly in the region that will become the AV junction is the first indication of AV cushion formation. The superior and inferior cushion, together known as the major AV cushions, are the first to form. They are found, respectively, on the ventral and dorsal wall (Figure 1B,D). In the mouse, this process starts around embryonic day (ED) 9.5. In these early stages, very few cells can be found in these cushions. Soon after, cell–cell interactions weaken in a subset of endocardial cells that line the cushions. This is followed by delamination and migration of these cells into the cushions where these endocardially-derived cells (ENDCs) then assume a mesenchymal phenotype. This process is referred to as endocardial-to-mesenchymal transition/transformation (endMT) [11,12].

A significant body of work has been conducted over the last few decennia that has led to the understanding that regulation of endMT is critically dependent on the unique molecular signature of the AV junctional myocardium as well as specific molecular characteristics of the AV endocardium [13,14,15]. The process of endMT is regulated by an intricate network of regulatory pathways and transcription factors and involves ligands and receptors of the TGFbeta superfamily of growth factors (e.g., TGFbeta2, BMP2 and BMPR1A), transcription factors (e.g., SOX9), and ligands and intermediates of the Notch, Hippo, and Wnt/beta-catenin signaling pathways [16,17,18,19,20,21,22,23,24]. Eventually, the ENDCs in the cushions become fibroblasts and are recognized in the maturing valves as (endocardially-derived) valve interstitial cells (endVICs). The fusion of the two major cushions in the midline of the common AV canal divides the canal into a separate left and right AV orifice [25].

The formation of the major AV cushions is followed by the formation of two lateral AV cushions. The lateral cushions, which are considerably smaller than the major cushions, develop on the lateral (or parietal) walls of the left and right myocardial AV junctions [25,26]. It is interesting to note that most of the experimental in vitro studies conducted to unravel the mechanism controlling endMT of the AV cushions have historically been performed using AV explants at stages in which the major AV cushions are developing, but before the onset of development of the lateral AV cushions. While, to the best of our knowledge, there are no data available to indicate that endMT in the lateral cushions is regulated by different mechanisms, the spatiotemporal difference in where and when they emerge remains to be resolved. Although not directly related to the development of the AV cushions and valves, it is worth noting that endMT is also responsible for the generation of mesenchymal cells that are found in the mesenchymal cap situated on the leading edge of the primary atrial septum [27,28,29,30,31]. Not much is known, however, regarding the mechanisms that control endMT in this structure [31].

## 3. Contribution of the AV Cushions to the AV Valves

For many years, it has been recognized that all four AV cushions are critical components for the development of the AV valves. In his work “Anatomie Menschlicher Embryonen III—Das Herz” (1885), Wilhelm His writes: “*An der Bildung der Atrioventricularklappen betheiligen sich einestheils die Muskelwand des Ohrkanales und des anstossenden Ventrikelgebietes, anderntheils die Bindesubstanzmasse der vier Atrioventricularlippen*” (freely translated: “*the tissues that contribute to the formation of the atrioventricular valves include the muscular wall of the (left and right) atrioventricular canals and the adjacent ventricular tissues, as well as the extracellular matrix of the four atrioventricular cushions*”). Our current understanding of cushion development is the result of more detailed descriptive and experimental studies and imaging techniques developed over the last 50 years or so. These new insights are the result of significant technological advances, development of experimental embryology, sophisticated in vivo and in vitro techniques, and breakthroughs in biochemistry, molecular biology, and, last but not least, transgenic mouse technology.

**Figure 2:**
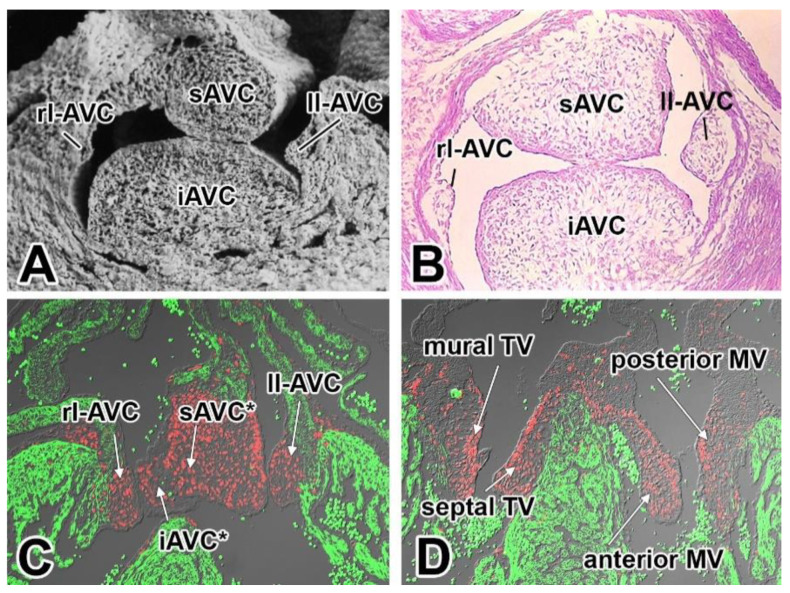
The AV cushions and their derivatives. A scanning electron microscopical (**A**) image and (**B**) a histological section show the major and lateral AV cushions at 13 ED. Immunofluorescently-stained sections of a 13.5 ED (**C**) and a 15 ED (**D**) heart show how the respective cushions contribute to the AV valve leaflets as the heart develops. The sections were stained for myosin heavy chain (green) and SOX9 (red). The asterisks in (**C**) are added to indicate that after the major cushions have fused, it is no longer possible to say with certainty whether a particular cell is derived from the iAVC or sAVC. Abbreviations: iAVC, inferior AV cushion; sAVC, superior AV cushion; ll-AVC, left lateral AV cushion; rl-AVC, right lateral AV cushion, MV, mitral valve; TV, tricuspid valve.

In the human heart, we distinguish two AV valves. The left AV valve (or mitral valve) will develop in the left AV junction, whereas, in the right AV junction, the right AV valve (or the tricuspid valve) will appear. The (fused) major AV cushions mainly contribute to the AV valve leaflets that are associated with the ventricular septum (Figure 1). The lateral AV cushions (Figure 1 and Figure 2A–C) contribute to the leaflets that are attached to the left and right atrioventricular junction. Histological analysis indicates that the superior AV cushion forms the “anlage” of the anterior (or aortic) leaflet of the mitral valve. The posterior (or mural) leaflet of the mitral valve, however, derives from the left lateral AV cushion. At the right side of the heart, the inferior AV cushion provides the bulk of the septal leaflet of the tricuspid valve (Figure 1D,E and Figure 2C,D). With subsequent development, the valve leaflets further mature and become organized in three layers: the atrialis, spongiosa, and fibrosa [32]. Each layer comprises a specific extracellular matrix composition and valve interstitial cells, which are essential for proper function of the leaflets. Given the fact that a significant amount of research on the development of the AV valves and investigations on the molecular pathways and pathogenic events that underlie the diseases of these valves are conducted in mice, it is important to recognize that, whereas the general mechanisms that lead to the formation of the valves are quite similar between mice and humans, the ultrastructure of the developed leaflets differs significantly. For instance, the valves in the human heart are, at their distal ends, contiguous with the papillary muscles via long and numerous tendinous chords (known as the chordae tendineae), while, in the mouse, these tendinous chords are not as prominent [25]. Furthermore, the right AV valve in the mouse does not have the typical three leaflet configuration as observed in the human, but rather consists of only two leaflets [33].

## 4. The Epicardium and Epicardially-Derived Cells (EPDCs)

The epicardium is a mesothelium-derived epithelium covering the myocardial surface of the heart. The development of the epicardium starts with the formation of the proepicardium, a morphological entity resembling a “cluster of grapes” located at the interface of the liver and sinus venosus at the cardiac venous pole [34,35,36,37,38]. The proepicardium is derived from a cardiac mesodermal progenitor pool by the cooperative interaction between BMP and FGF signaling [39,40]. After the proepicardium has formed, cells detach from the proepicardium and adhere to the “naked” myocardial surface of the looping heart, where they spread as an epithelium over the developing cardiac compartments [38,41]. Subsequently, an ECM-rich space develops between the myocardium and epicardium. This space is known as the subepicardium. In a process similar to what we see in the AV cushions, an epicardial-to-mesenchymal transformation (epiMT) results in the generation of epicardially-derived cells (EPDCs) [10]. Whereas historically, the epicardium was first and foremost seen as a protective layer and allowing for smooth movement in the pericardial environment during the cardiac cycle, a series of studies published over the last 20 years has convincingly demonstrated that the epicardium plays a far more important role in the development and function of the heart than previously thought. This insight developed as a result of early cell fate studies using quail-chick chimeras [10,35,42] as well as other labeling approaches using experimental embryology with molecular marking techniques [43]. These studies demonstrated that EPDCs can cross the boundary between the subepicardial space and the adjacent myocardium and can subsequently migrate into the myocardial walls. Moreover, these and other studies have shown that, after populating the myocardial walls, the EPDCs can differentiate into interstitial fibroblasts, pericytes, coronary smooth muscle cells, and coronary endothelium [10,42,43,44,45,46]. In a number of these cell-fate tracing studies, it was suggested that EPDCs contributed to the developing AV valves [10,35,42]. In the context of this contribution, we will concentrate on the role that EPDCs play in the development of the tissues at the AV junction, and, in particular, the AV valves.

## 5. The Formation of EPDCs at the AV Junction (AV-EPDCs)

Whereas tracking the fate of EPDCs with various avian model systems did reveal significant new insights in the potential role of the epicardium in heart development [10,35,42,43], the complex experimental nature of the microsurgical manipulations, as well as concerns about the biological relevance of some of the approaches (e.g., would cells from an explanted proepicardium isolated from a quail and transferred to a chick embryo truly recapitulate the behavior of endogenous chick epicardial cells, etc), prompted the development of different approaches allowing the study of EPDCs in the developing mammalian heart. The emergence of the cre-lox mouse technology [47] opened new avenues to explore the role of the epicardium in the mouse. Based on the epicardial-specific expression of several transcription factors (e.g., TBX18 and WT1), a number of “epicardial-specific” cre-mouse models were developed that allowed the labeling of the epicardium and fate tracing of EPDCs during development. In our published and ongoing studies, we use the mWt1/IRES/GFP-Cre mouse (referred to as mWt1^Cre^) in combination with the B6.129(Cg)-Gt(ROSA)26Sor^tm4(ACTB-tdTomato,-EGFP)Luo^/J (referred to as R26^mT/mG^) reporter mouse [46]. Using this model (in this paper, referred to as the Wt1^Cre^;R26^mT/mG^ model), we have conducted cell fate studies and are continuing to investigate the importance of AV-EPDCs in the development of the AV junction by targeting molecular pathways that we consider to be important for epicardial cell biology [48,49].

**Figure 3:**
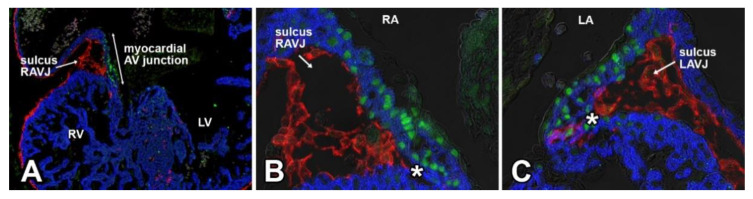
Epicardially-derived cells in the AV sulcus at ED12. EGFP-labeled AV-EPDCs (pseudo-colored in red) are providing the mesenchyme of the AV sulcus. Staining for the transcription factor TBX3 (green) delineates the AV-junctional myocardium (**A**–**C**). As demonstrated in (**A**–**C**), the AV-EPDCs penetrate the myocardial wall (blue) at the lower boundary of the AV-junctional myocardium. (**B**) is a higher magnification of (**A**).

While the generation of EPDCs through epiMT takes place in all regions of the epicardium covering the chamber myocardium, it is more prevalent in the epicardium lining the ventricles than the epicardium on the atria. EpiMT is particularly prominent in the epicardium found at the AV junction. Here, the accumulation of extracellular matrix (ECM) and AV-EPDCs (as we will call this specific population of EPDCs in the AV junction in the rest of this contribution to differentiate them from the EPDCs in other parts of the heart) leads to the formation of the AV sulcus (Figure 3). As mentioned, the molecular regulation responsible for the formation of AV-ENDCs in the AV cushions has been studied in detail. The formation of AV-EPDCs in the AV sulcus has, however, not been studied to the same extent. As described above, the myocardial AV junction plays a crucial role in the regulation of endMT in the AV cushions. While there are many regulatory mechanisms involved in this process, TGFbeta2 [50,51] and BMP2 [18,52], in particular, are of particular importance as both are expressed at a relatively high level in the AV myocardium. In one of the following sections, we will come back to the role of growth factor signaling and epicardial development.

## 6. Contribution of AV-EPDCs to the Developing AV Valves

In 2012, we published an epicardial cell-fate study using the Wt1^Cre^;R26^mT/mG^ model. Part of the study was focused on the role of AV-EPDCs in the developmental events at the AV junction during valvuloseptal development [46]. We described how, after the onset of formation of the AV sulcus, the AV-EPDCs that have been generated through epiMT migrate toward the myocardial AV junction and then penetrate the junction at the lower boundary of the AV myocardium. As a result, the embryonic AV myocardium becomes incorporated into the lower margin of the atrial chambers (Figure 3). Importantly, it also leads to the formation of the annulus fibrosus, which, in the adult heart, is a ring of connective tissue that physically and electrically insulates the working myocardium of the atria from that of the ventricles, thus preventing ventricular pre-excitation during atrial activation [46,53]. Subsequent to the formation of the annulus fibrosus, a cohort of AV-EPDCs migrates into the lateral AV cushions, i.e., the cushions that participate in the formation of the parietal AV valve leaflets [46,54]. This process starts around ED12. As the lateral cushions further develop into the parietal leaflets of the left and right AV valves, the number of AV-EPDCs in these leaflets gradually increases and large numbers of AV-EPDCs are found in the parietal leaflets even after birth. These observations challenged the long-held belief that (virtually) all cells in the developed AV valves derive from the endocardial cell lineage [55].

**Figure 4:**
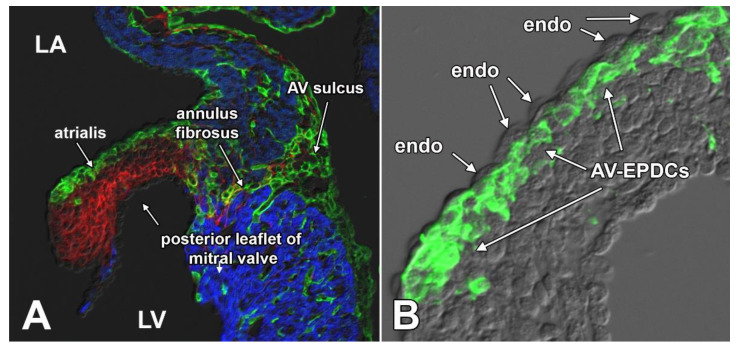
AV-EPDCs migrate into and occupy the subendocardial space at the atrial side of the left parietal leaflet (posterior leaflet of mitral valve). This figure shows the left AV junction of a ~16 ED Wt1^cre^;R26^mG^ heart. The section shown in (**A**) was stained for myosin heavy chain (MF20, blue), EGFP expression to delineate EPDCs (green), and periostin (red). Panel (**B**) shows a detail of the leaflet, only showing the EGFP expression, demonstrating that the EGFP-positive AV-EPDCs are located immediately below the endocardial lining of the leaflet. LA, left atrium; LV, left ventricle; endo, endocardium.

A specific feature associated with the contribution of AV-EPDCs into the cushions is that they position themselves right underneath the layer of valve endocardial cells that lines the atrial side of the developing leaflet. This part of the leaflet is, specifically in the human, typically referred to as the atrialis [32] (Figure 4).

**Figure 5:**
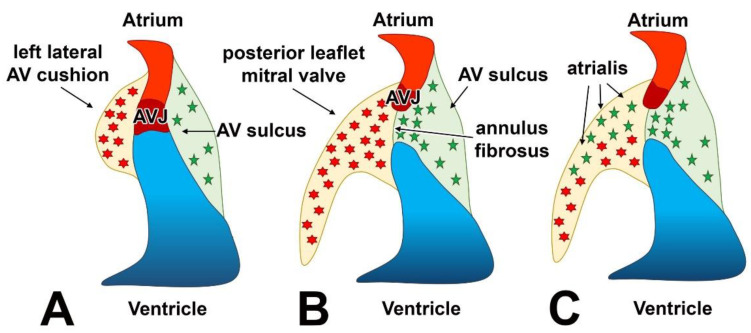
Schematic depiction of the epicardial contribution during the development of the left AV junction. Panel (**A**) shows that at early stages of development, the atrial and ventricular myocardium are contiguous through the AV-junctional myocardium. End-MT generates ENDCs (red cells) in the left lateral AV cushion, while epiMT is responsible for the formation of AV-EPDCs (green cells). Panel (**B**) depicts the situation in which AV-EPDCs have formed the annulus fibrosus. In panel (**C**), it is demonstrated that the AV-EPDCs start to populate the lateral AV valve leaflet (i.e., the posterior leaflet of the mitral valve).

The contribution of AV-EPDCs to the developing tissues at the AV junction is schematically depicted in Figure 5.

At this point, we have not yet elucidated the mechanisms that control the directional migration of the AV-EPDCs into the parietal leaflets. It is, however, in the context of this special review, of interest to note that unpublished/preliminary results have identified a few candidate mechanisms that we are currently pursuing.

**Figure 6:**
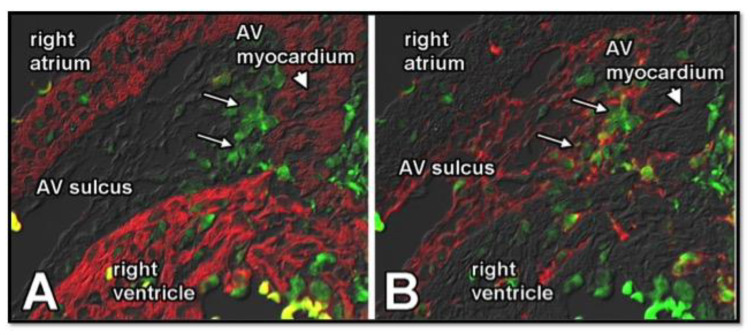
MMP2 expression in AV-EPDC in the AV sulcus. Sister sections of a Wt1^cre^;R26^mG^ heart were stained for MMP2 (green in (**A**,**B**)), myosin (red in (**A**)), and EGFP (to delineate EPDCs, red in (**B**). The figure shows that MMP2 is upregulated in the AV-EPDC as they are migrating toward the AV myocardium.

MMP2 and Type IV Collagen: As has been published previously, migrating EPDCs in the AV junction express Matrix-specific Metalloproteinase 2 (MMP2) [53]. Our preliminary data show that MMP2, which is also known as type IV collagenase, becomes upregulated in the AV-EPDCs as they move from the epicardial surface toward the AV myocardium before they invade the AV junction (Figure 6). This suggests a role for MMP2 in the migration of AV-EPDCs into the junctional myocardium and into the AV valves. As one of the major known functions of MMP2 is to cleave type IV collagen, we decided to investigate whether type IV collagen could potentially be a target for MMP2 in the developing heart valves. Immunostaining for type IV collagen of hearts at stages of active AV-EPDC migration showed that this collagen isoform is expressed in the region under the endocardial lining at the atrial aspect of the leaflets, i.e., the area through which the AV-EPDCs migrate as they start to populate the parietal leaflets. This observation suggests that the valve endocardial cells produce type IV collagen and makes it plausible that the collagenase activity of MMP2 in the AV-EPDCs facilitates the migration of these cells by degradation of type IV collagen.

**Figure 7:**
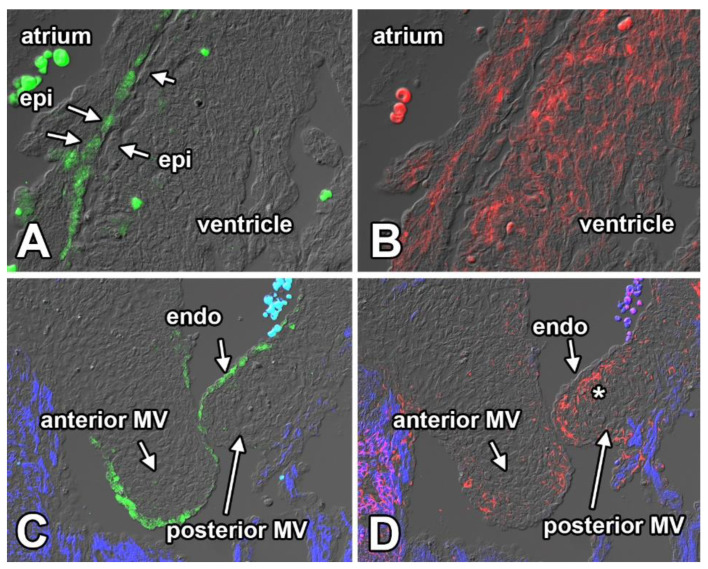
α4 Integrin and VCAM-1 expression. Immunostained sections of a 13.5 ED heart labeled for α4 Integrin (green in (**A**,**C**)) and VCAM-1 (red in (**B**,**D**)) and myosin heavy chain (blue in (**C**,**D**)). The figure shows expression of α4 Integrin in the atrial epicardium (**A**) and valve endocardium (**C**) and expression of VCAM-1 in atrial and ventricular myocardium (**B**) and valve mesenchyme (asterisk in (**D**). endo, endocardium; epi, epicardium.

VCAM-1 and α4 Integrin: Migration is an integrated process requiring temporary interactions between environmental substrates and receptors on the migrating cells. The cell surface receptor α4 integrin mediates cell–extracellular matrix (ECM) and cell–cell adhesion by interacting with vascular cell adhesion molecule 1 (Vcam-1) [56,57]. Migration of epicardial cells over the surface of the myocardium is known to be critically dependent on the expression of the α4 integrin on the epicardial cell surface and expression of Vcam-1 in the myocardium. Mice that lack either α4 integrin or Vcam-1 fail to develop an epicardium and die in early embryonic stages [57,58]. To determine whether α4 integrin and Vcam-1 could also be involved in migration of AV-EPDCs in the valves, we conducted expression studies on ED13.5 and ED14.5 mouse embryos. The immunolabeling confirmed α4 integrin expression on a subset of epicardial cells, the expression being strongest in the epicardial cells plastered against the atrial myocardium (Figure 7A). We saw little α4 integrin expression in epicardially-derived cells, including AV-EPDCs, suggesting that EPDCs lose their α4 integrin expression after undergoing epiMT. Surprisingly, expression of α4 integrin was found in the endocardial lining of the AV valve leaflets (Figure 7C). Immunolabeling for Vcam-1 showed, as expected, expression in atrial and ventricular myocardium (Figure 7B). Vcam-1 expression was also observed in valve mesenchyme, specifically in cells in close spatial association with the endocardium (Figure 7D). While these observations merely demonstrate a spatial relationship between the expression of α4 integrin and Vcam-1 and the location where AV-EPDCs eventually become localized, we believe it merits further investigation on the role of these factors in the migration of AV-EPDCs into the AV junctional tissues and the parietal leaflets of the AV valves.

## 7. Molecular Control of Epicardial Development at the AV Junction

Insight into the molecular regulation of epiMT in general has, over the years, largely been obtained from in vitro studies using chick and mouse epicardial explants and from studies using primary epicardial cell cultures and immortalized epicardial cell lines [59,60,61,62,63,64,65]. Combined, these studies have identified several factors implicated in the process, including the transcription factors WT1 [66], SNAIL and SLUG, growth factors such as BMP2 [61], TGFβ1 [67] and TGFβ2 [68], and cell surface receptors, including PDGFRα [65], PDGFRβ [65], TGFβR1/Alk5 [68], TGFβR3 [60,61], and BMPR /ALK3 [48,61]. Molecular analysis of isolated EPDCs from the AV regions of 14.5 ED hearts showed that some genes identified in the aforementioned studies were expressed in/by the AV-EPDC, including SMAD1, SNAIL and SLUG, factors involved in EMT (epiMT in this case), genes that are typically associated with fibroblast differentiation, such as periostin, procollagen I, fibronectin I, vimentin, discoidin domain receptor 2 (DDR2), and tenascin C, as well as matrix metalloproteinase 2 (MMP2), an enzyme that is important in the regulation of cell migration [53]. In a more recent paper, the significance of the PRMT1-p53 pathway in epiMT, the migration of EPDC into the ventricular myocardial walls and the formation of coronary vessels has been reported [69]. The specific role of this pathway at the AV junction, however, remains to be determined.

When focusing on AV-epiMT, it is important to note that, of the growth factors mentioned, only BMP2 and TGFβ2 are expressed at high levels within the AV myocardium [18,48,70,71,72]. The expression of these two secreted growth factors has been proven to be of critical importance for the induction and regulation of endMT in the AV cushions which are located at the luminal side (or inside) of the myocardial AV junction. Based on these considerations, we decided a few years ago to investigate the role of BMP signaling in the AV epicardium [48]. To determine the importance of BMP signaling for epicardial-dependent development of the AV junction, we created the WT1^cre^;ALK3^fl/fl^ mouse, which allowed us to specifically delete ALK3 from the epicardial lineage [48,49]. When crossed with the ROSA26^mT/mG^ mouse [73] (WT1^cre^;ALK3^fl/fl^;R26^mG^), it enabled us to follow the fate of the EPDCs in the absence of ALK3. In this current review, we will highlight the results as they relate to the development and maturation of the AV valve leaflets and discuss the significance of the observations in the context of the pathogenesis of myxomatous valve disease (MVD).

**Figure 8:**
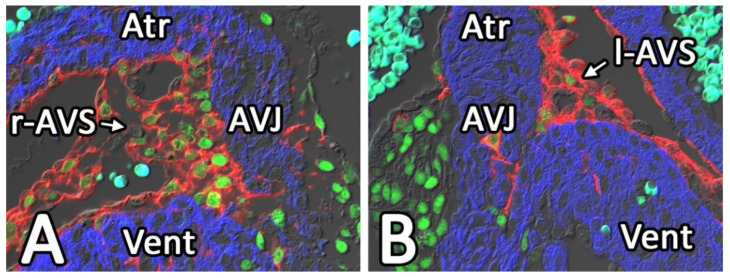
SOX9 is upregulated in AV-EPDCs as they migrate from the epicardium toward the right (**A**) and left (**B**) AV junction. A section of a 13 ED WT1^cre^;R26^mT/mG^ heart was stained for SOX9 (green), EGFP (immunolocalized and visualized in the red channel) to localize EPDCs, and the myocardium (myosin heavy chain) in blue. Atr, atrium; AVJ, (myocardial) AV junction; l-AVS, left AV sulcus; r-AVS, right AV sulcus; Vent, ventricle.

The transcription factor SOX9 has been implicated in the regulation of endMT, in controlling proliferation of ENDCs [74,75,76], and plays an essential role in the development of the endocardial cushions [77,78,79,80]. Its expression is regulated by BMP2. Just like BMP2, SOX9 is also involved in the transcriptional regulation of the expression of ECM components in developing cardiac structures, such as Cartilage Link Protein (Crtl1) and Tenascin [74,75,76]. In the epicardium, SOX9 is expressed at a relatively low level. The importance for SOX9 in the ventricular epicardium was demonstrated in studies that showed that reduced SOX9 expression in an epicardial-specific PDGFR knockout mice led to the loss of epicardial cell migration and reduced epiMT [65]. As described in our earlier studies [46], co-labeling for EGFP and SOX9 in WT1^cre^;R26^mT/mG^ specimens shows prominent expression of SOX9 in the EGFP-positive AV-EPDCs within the AV-sulcus (Figure 8). As SOX9 is a known target for BMP2, this strongly suggests that the elevated expression levels of BMP2 in the AV myocardium is responsible for the upregulation of SOX9 in the AV-EPDCs. SOX9 expression is also observed in the AV-EPDCs that form the annulus fibrosus and in the AV-EPDCs that populate the AV valve leaflets that derive from the lateral AV cushions.

## 8. Significance of Epicardial ALK3 and SOX9 Expression in AV Valve Development

**Figure 9:**
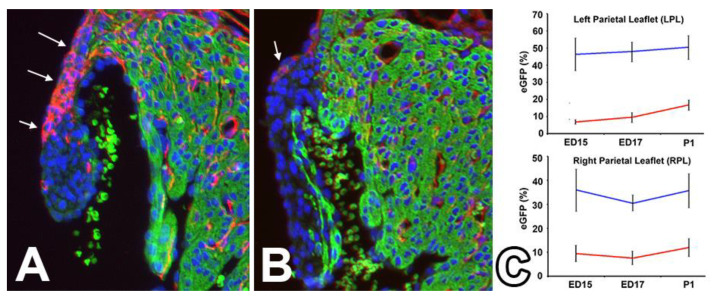
Contribution of EPDCs to the posterior leaflet of the mitral valve of control (**A**) and WT1^cre^;ALK3^fl/fl^;R26^mG^ (**B**) hearts at ED17. The sections in panels (**A**,**B**) were stained for EGFP (immunolocalized and visualized in the red channel) to localize EPDCs, the myocardium (myosin heavy chain in green), and DAPI to visualize all nuclei. Panel (**C**) shows a graph with the percentage of EGFP-positive cells (determined using AMIRA 3D software), in the left and right parietal leaflets of ED15, ED17, and postnatal day 1 (P1) hearts. The blue line represents percentage of EGFP-positive cells in control hearts, the red line represents this percentage in the heart of WT1^cre^;ALK3^fl/fl^;R26^mG^ specimens (adapted from Lockhart et al., 2014).

As shown in our earlier study [46], AV-EPDCs are responsible, in part, for the formation of the AV sulcus and annulus fibrosus and contribute significantly to the cellular content of the parietal AV valve leaflets in late fetal and neonatal hearts. Deletion of ALK3 from the epicardial cell lineage using the WT1^cre^;ALK3^fl/fl^ model resulted in failure of the AV sulcus and annulus fibrosus to properly develop and led to a significant reduction in the number of AV-EPDCs in the parietal leaflets. In control hearts between ED15 and day one after birth (P1), AV-EPDCs occupy up to 50% of the total cell population in the left and right parietal AV valve leaflets, whereas, in the WT1^cre^;ALK3^fl/fl^;R26^mG^ mice, that number fell to less than 20% (Figure 9). An interesting, and unexpected, finding was that the overall number of cells within the leaflets did not significantly change, an observation from which we concluded that the number of cells with a non-epicardial origin was increased. Although this still formally needs to be proven, we believe that these non-epicardially derived cells are mostly AV-ENDCs. When the studies that led to this publication on the WT1^cre^;ALK3^fl/fl^ model were conducted, there were no tools available to confirm that this was the case. New developments in the generation of mouse models that facilitate the tracing of multiple cell populations within one specimen (see, for instance, [54]) will allow us to test this hypothesis. Interestingly, we did not observe a significant increase in the level of proliferation of the non-EPDCs. As we believe that the non-EPDCs cells are endocardially-derived, the increase in their number could point at a higher rate of endMT in the absence of the AV-EPDCs. In this context, it is important to reiterate that as they migrate into the lateral cushions/developing parietal valve leaflets, the AV-EPDCs position themselves right under the endocardium on the leading edge of the cushions (see Figure 4). Our current working hypothesis is that, by positioning themselves between the AV myocardium and the endocardium, AV-EPD Cs play a role in controlling endMT. In the absence/reduced presence of AV-EPDCs, this control may be lifted, resulting in an increase in endMT. Studies to test this hypothesis are ongoing.

**Figure 10:**
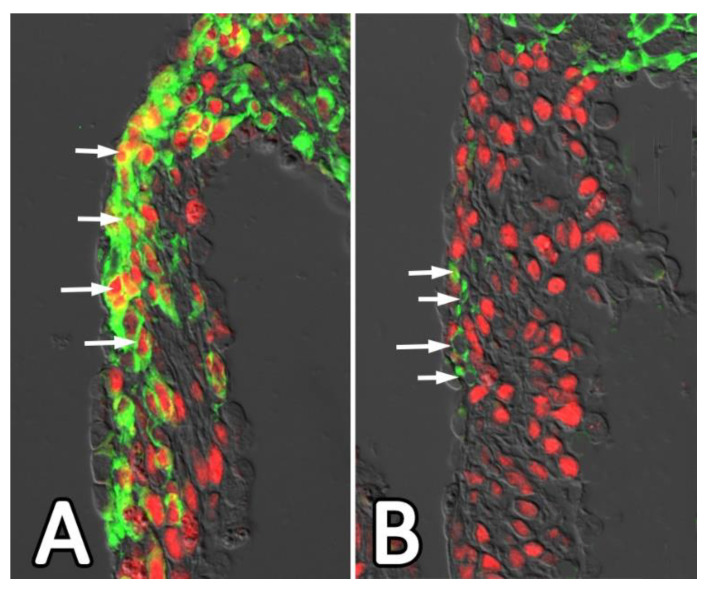
AV-EPDCs (green) in posterior leaflet of the mitral valve in control (**A**) and WT1^cre^;SOX9^fl/fl^; R26^mG^ (**B**) specimens at 17.5 ED. The sections are stained for the presence of EGFP (in green), indicating the presence of EPDCs and SOX9 (red). Note the abundance of SOX9-positive AV-EPDCs in the leaflet of control heart (arrows in (**A**)) and virtual absence of AV-EPDCs in the posterior leaflet of the knockout heart (**B**). The few AV-EPDCs that are found in this leaflet do not express SOX9 (arrows in (**B**)). Note also that EGFP-positive EPDCs in the top right corner of (**B**) do not express SOX9.

To determine the role of SOX9 in the epicardial-dependent development of the AV junction, we generated the WT1^cre^;SOX9^fl/fl^ model. Our preliminary and unpublished results from this ongoing study indicate that removing SOX9 from the epicardial lineage has a moderate effect on the development of the AV sulcus when compared to what was observed in the WT1^cre^;ALK3^fl/fl^ mouse. This may indicate that epiMT is not affected to the same degree as observed in the WT1^cre^;ALK3^fl/fl^ mouse but this will remain to be established. The impact on the migration of AV-EPDCs into the parietal leaflets of the AV valves, including the posterior leaflet of the mitral valve as demonstrated in Figure 10, however, is pronounced. This suggests that SOX9 is involved in migration of AV-EPDCs in post-epiMT stages. In an earlier study, in which the importance for SOX9 in the ventricular epicardium was investigated, it was shown that reduced SOX9 expression in epicardial-specific PDGFR knockout mice not only resulted in a reduction in epiMT, but also a loss of epicardial cell migration [65]. Our findings on the behavior of the EPDCs at the AV junction in the WT1^cre^;SOX9^fl/fl^ mouse are, therefore, compatible with the reported role of SOX9 in EPDCs in other parts of the heart.

## 9. Consequence of Reduced Presence of AV-EPDCs in the Valve Leaflets—A Link to Myxomatous Valve Disease

**Figure 11:**
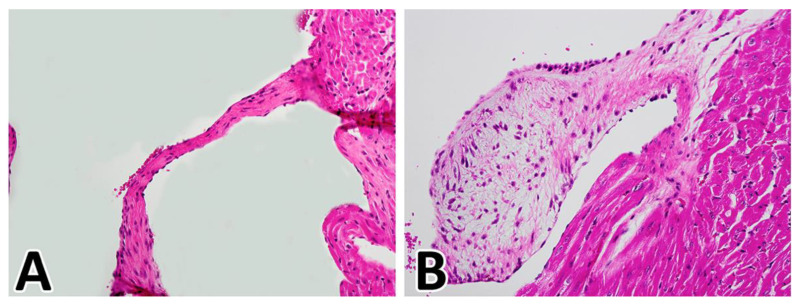
Myxomatous Valve Degeneration in the WT1^cre^;ALK3^fl/fl^ mouse. Panel (**A**) shows H/E staining of the posterior leaflet of the mitral valve of a control heart, panel (**B**) shows the H/E staining of the myxomatous leaflet in a WT1^cre^;ALK3^fl/fl^ littermate collected 10 days after birth.

In our study on the WT1^cre^;ALK3^fl/fl^ mouse [48], we found that even though the overall cellular composition of the leaflets significantly changed as a result of the deletion of ALK3 from the epicardial cell lineage, the morphology of the parietal leaflets remained remarkably normal throughout fetal development. Histological analysis of post-natal mitral valve morphology of WT1^cre^;ALK3^fl/f^ and control littermates up to 20 weeks after birth showed, however, that the valve leaflets, and, in particular, the posterior leaflet of the mitral valve, were significantly enlarged. Anatomically, these leaflets resembled AV valve leaflets as seen in human patients with MVD/MVP [81] (Figure 11). Myxomatous degeneration of AV valves in humans and mouse models for MVD is typically associated with increased expression of proteoglycans and glycosaminoglycans (GAGs) [3,82,83]. To determine the level of expression of GAGs and proteoglycans in the postnatal WT1^cre^;ALK3^fl/fl^;R26^mG^ heart, we conducted immunolabeling for versican-β and hyaluronan. In control hearts, versican-β and hyaluronan were found in the distal tip of the parietal leaflets, while, in the enlarged leaflets of WT1^cre^;ALK3^fl^***/***^fl^;R26^mG^ specimens, large amounts of hyaluronan and versican-β were detected throughout the leaflets, consistent with a myxomatous phenotype as seen in humans [48].

**Figure 12:**
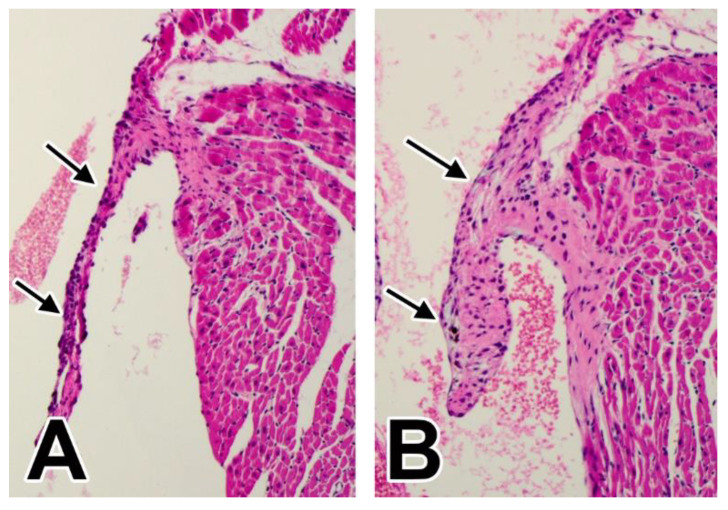
Myxomatous posterior leaflet in a WT1^cre^;SOX9^fl/fl^ specimen at one month after birth. Panel (**A**) shows an H/E staining demonstrating the posterior leaflet of a control heart, panel (**B**) shows the myxomatoyus phenotype of the posterior mitral valve leaflet, in a WT1^cre^;SOX9^fl/fl^ specimen.

Given the fact that the reduction in contribution of the AV-EPDCs to the developing valves of the WT1^cre^;SOX9^fl/fl^;R26^mG^ mouse resembled what we have seen in the WT1^cre^;ALK3^fl/fl^;R26^mG^ mouse, we sought to determine whether the reduction in AV-EPDCs in the valve leaflets would also lead to valve abnormalities later in life. Neonatal WT1^cre^;SOX9^fl/fl^;R26^mG^ specimens and mice at one and two months of age were collected and analyzed. Like the WT1^cre^;ALK3^fl/fl^;R26^mG^, the WT1^cre^;SOX9^fl/fl^;R26^mG^ were also found to develop MVD (Figure 12).

Given all of the above, we believe that the pathological features of the valves are, in all likelihood, the direct consequence of the reduced presence of the AV-EPDCs in these valves. How this reduction in the number of AV-EPDCs exactly affects the cell biology and behavior of the non-EPDCs that contribute to valve development and function remains to be determined. The main question generated by these studies is whether “developmental” defects that interfere with the normal epicardial contribution to the leaflets of the AV valves should be considered when investigating the pathogenesis of MVD in the human population.

## 10. Discussion and Reflection

Dr. Adriana Gittenberger-de Groot (“Adri” for friends and colleagues) was one of the major driving forces in the field of cardiovascular developmental biology. Her first publication dates from 1971 [84] and, for nearly 50 years, she contributed to our current understanding of the development of the heart and the pathogenesis of congenital heart defects. In 1998, she was the lead author on a paper titled *“Epicardium-derived cells contribute a novel population to the myocardial wall and the atrioventricular cushions”* [10], a publication that, together with a few other articles that appeared around the same time [42,43,45], revolutionized our insight into the role of the epicardium in heart development. These studies truly formed the foundation for all the subsequent studies in which the contribution of EPDCs to the developing mouse heart has been studied (mainly using the cre-lox system) and the studies in which the molecular regulation of epicardial development is investigated. Together with her colleagues at Leiden University, Adri has also significantly contributed to many other aspects of heart development, including to our growing understanding of atrioventricular valve development [85,86] and the pathogenesis of congenital heart disease at times when words like PCR, cre-lox mouse models, and confocal microscopy, were not yet part of the developmental biologist’s vocabulary. In this review, we have presented some of our published and ongoing work on aspects of heart development and pathogenesis that, directly and indirectly, can be associated with the scientific legacy that Adri has left behind. The possibility that our work shows that there might be a developmental origin for (some forms of) MVD is intriguing. Even without a link to valve pathogenesis, the “how” and “why” of the directional migration of AV-EPDCs into the parietal AV valve leaflets, and the role that these cells might play in valve development and function, are topics that need significant attention in the years to come and that will further our insight into the complexity of valve formation.
